# Noninvasive Electroencephalogram Based Control of a Robotic Arm for Writing Task Using Hybrid BCI System

**DOI:** 10.1155/2017/8316485

**Published:** 2017-06-01

**Authors:** Qiang Gao, Lixiang Dou, Abdelkader Nasreddine Belkacem, Chao Chen

**Affiliations:** ^1^Key Laboratory of Complex System Control Theory and Application, Tianjin University of Technology, Tianjin 300384, China; ^2^Endowed Research Department of Clinical Neuroengineering, Global Center for Medical Engineering and Informatics, Osaka University, Osaka 565-0871, Japan

## Abstract

A novel hybrid brain-computer interface (BCI) based on the electroencephalogram (EEG) signal which consists of a motor imagery- (MI-) based online interactive brain-controlled switch, “teeth clenching” state detector, and a steady-state visual evoked potential- (SSVEP-) based BCI was proposed to provide multidimensional BCI control. MI-based BCI was used as single-pole double throw brain switch (SPDTBS). By combining the SPDTBS with 4-class SSEVP-based BCI, movement of robotic arm was controlled in three-dimensional (3D) space. In addition, muscle artifact (EMG) of “teeth clenching” condition recorded from EEG signal was detected and employed as interrupter, which can initialize the statement of SPDTBS. Real-time writing task was implemented to verify the reliability of the proposed noninvasive hybrid EEG-EMG-BCI. Eight subjects participated in this study and succeeded to manipulate a robotic arm in 3D space to write some English letters. The mean decoding accuracy of writing task was 0.93 ± 0.03. Four subjects achieved the optimal criteria of writing the word “HI” which is the minimum movement of robotic arm directions (15 steps). Other subjects had needed to take from 2 to 4 additional steps to finish the whole process. These results suggested that our proposed hybrid noninvasive EEG-EMG-BCI was robust and efficient for real-time multidimensional robotic arm control.

## 1. Introduction

Brain-computer interface (BCI) offers a direct communication channel between the brain and external devices without relying on the brain normal output pathways of peripheral nerves and muscles [1]. According to different signal acquisition methods, BCI systems can be divided into two main categories, invasive and noninvasive BCI systems [2–5]. Although invasive BCI systems seem suitable for some clinical applications because of their high signal-to-noise ratio (SNR) and the information transfer rates (ITR), they still suffer from potential surgical risks and postoperative immune response. Noninvasive BCIs might be more suitable for daily life applications for many socioeconomic reasons such as the user's safety and a relatively low cost. Electroencephalography (EEG) is a popular electrophysiological monitoring method to record brain activity and is widely used in noninvasive BCI researches and applications since Berger's discovery [6] and Vidal's first BCI prototype [7].

Nowadays, there are huge opportunity and necessity for helping handicapped people to enhance or increase their abilities to interact with complex environment, such as rehabilitation training sessions, mind-controlled prosthetic arm applications [8, 9], and augmentative and alternative communication systems [10]. However BCIs can also benefit healthy people for entertainment and increasing their independency, especially for elderly persons [11–13]. For decades, several EEG-based typical BCI systems have been proposed based on slow cortical potential (SCP) [14], motor imagery (MI) [15], steady-state visual evoked potential (SSVEP) [16], and the P300 wave of the human event-related potential [10, 17]. Each type of these BCI systems has its unique advantages and some disadvantages. For example, the SSVEP-based BCI system has many advantages such as less training and higher SNR and ITR. MI-based BCI has the advantage of fast response but is limited by the number of tasks. Therefore, there has been increasing interest in solving dimensionality issue by using hybrid BCI which has to be composed of two BCI modalities' combination (e.g., motor imagery with P300, motor imagery with SSVEP, and P300 with SSVEP) or it can be also a combination of brain and nonbrain activity such as eye movements (EOG), muscles activity (EMG), and heart electrical activity (ECG) to improve the overall performances of BCI systems [18–22]. The electrical activity of muscles can easily interfere with EEG signal considering the anatomical locations of facial or masticatory muscles surrounding the skull. This myogenic contamination of the EEG can constitute a serious problem in BCI applications and it can be useful information in the same time for developing hybrid BCIs. Pfurtscheller et al. proposed an online system using SSVEP-based BCI and a type of ERD BCI, called a “brain switch” [19]. Lately, Punsawad et al. controlled practical machine through hybrid EEG-EOG brain-computer interface system [23]. Then Wang et al. controlled wheelchair directions through unilateral hand imagination and a wheelchair speed through P300 and EOG [24].

Controlling a robotic arm with noninvasive hybrid BCIs surely provides a desirable alternative, but prior to this study it has not been shown that such hybrid systems could achieve multidimensional control of robotic arm in three-dimensional (3D) space. These systems offer a potentially effective control for complex and naturalistic environment through the combination of brain- and nonbrain-based multifunctional BCI. They can reduce user fatigue by switching from a modality to another and increase the degree of freedom for augmentative and alternative BCI systems. The aim of this study is to improve the performance of BCI system by design a new hybrid EEG-EMG-BCI system (i.e., combination of brain activity (MI and SSVEP) with muscles activity such as teeth clenching). In this paper, a hybrid BCI system was described, including motor imagery-based brain switch, “teeth clenching” state detector, and a steady-state visual evoked potential- (SSVEP-) based BCI. In our proposed hybrid BCI, motor imagery decoding was used as a single-pole double throw brain switch (SPDTBS) which can complete multitasks, combined with 4-class SSVEP-based BCI system. In addition, “stop” command was executed by recognizing facial action by recording EMG artifact from EEG signals. For real-time application, a writing task was implemented to verify the performances of our proposed hybrid system. Healthy subjects succeed to write an English word though our proposed hybrid noninvasive BCI system. In the following sections, we describe our proposed system in detail and its real-time writing application to enhance the user's abilities to interact with a complex environment.

## 2. Methods

### 2.1. Experimental Paradigm

**Figure 1:**
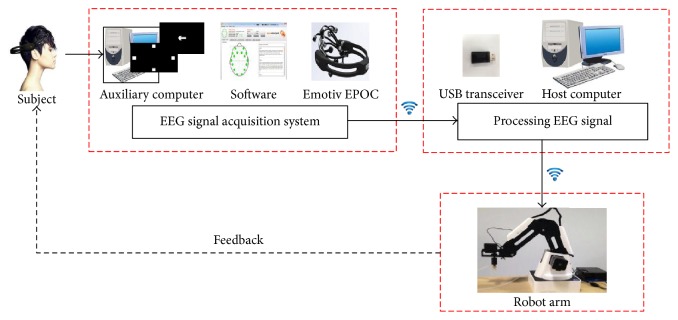
Schematic architecture of the experimental setup for the real-time hybrid BCI-controlled robotic arm.

Our proposed hybrid noninvasive EEG-EMG-BCI system mainly consists of three hardware components which are a portable EEG acquisition device (Emotiv EPOC), a host computer, and a robotic arm (see Figure 1). The EEG signals were recorded and transmitted to the host computer with an USB transceiver dongle. Then, EEG signals were processed and decoded in the host computer. Based on this proposed BCI architecture, the intension of subjects was transformed to multidimensional control commands and sent to operate the Dobot (robotic arm) via the wireless module in real time.

**Figure 2:**
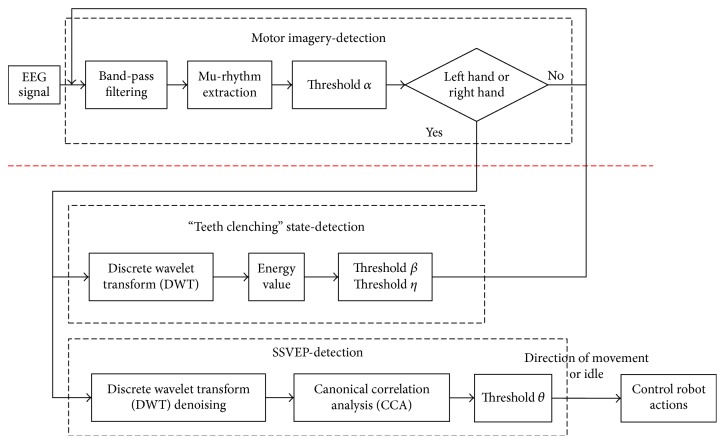
Flowchart of the proposed algorithm for hybrid EEG-EMG-BCI system.

The hybrid BCI consists of MI-, EMG-, and SSVEP-based BCI systems. As shown in Figure 2, the subject imagines the left hand or right hand movement for 4 s as the first step. Once the imagined movements' type (e.g., left hand and right hand movements) was confirmed, the system will enter the second hybrid BCI phase. In the second phase, SSVEP and “teeth clenching” were decoded. The presence of “teeth clenching” was detected during the second phase. Once “teeth clenching” state is confirmed, the program will execute stop command of SSVEP modality and go back to the first motor imagery modality. For SSVEP paradigm, four white blocks (with different frequencies: 6 Hz, 7.5 Hz, 8.57 Hz, and 10 Hz) of stimuli flicker were presented at the top, bottom, left, and right positions in black board.

**Figure 3:**
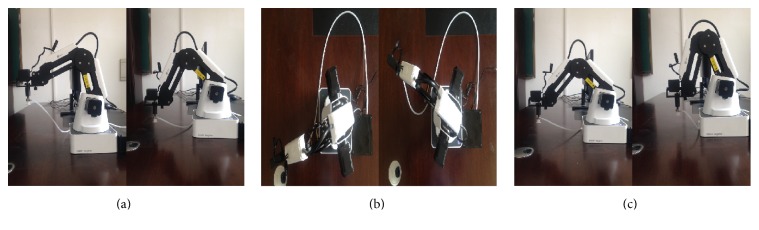
The six possible directions of the robotic arm. (a) Upward and downward movements. (b) Left and right movements. (c) Forward and backward movements.

According to each unilateral movement (right or left hand imagination), the SPDTBS was designed. The SPDTBS was combined with four tasks of SSVEP-based BCI modality to provide more commands (i.e., to achieve multidimensional BCI control) for the robotic arm movements such as the forward, backward, left, right, upward, and downward movements (see Figure 3). All these commands were shown in Table 1.

**Figure 4:**
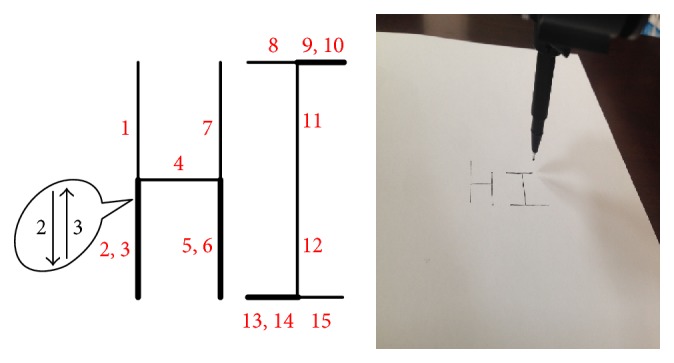
Essential steps for the robotic arm to write the word “HI” with the writing result of the robotic arm controlled by our proposed hybrid BCI in the right side.

Eight healthy subjects participated in the experiment (age 23.62 ± 1.06 years (mean ± standard deviation “SD”); one female and seven males). All of them were undergraduate students, without any experience with BCI system. The subjects were seated in a comfortable chair, 50 cm away from the computer screen. The robot arm was placed on the table, about 45° in the left front of the subject. Each subject was able to look at both the monitor and the movement of the robot arm. The subjects were requested to write the word “HI” and the essential steps were shown in Figure 4. It takes at least 15 steps to complete the writing of BCI; each step represents a horizontal or vertical line. The subjects can choose the order of writing, and each step can be written repeatedly.

The following points are used to evaluate the performance of the hybrid BCI system:

(1) Time: time required to complete a task.
(2) Step count: the number of steps to complete the task in paper.
(3) Obvious errors: number of obvious errors. For example, if the writing task is O letter but the result is Q, this result is defined as obvious error.
(4) Information transfer rate, which is defined as
  (1) ITR=60T×log2⁡N+P log2⁡P+1−Plog2⁡1−PN−1,
where *N* is the number of targets, *P* is the accuracy rate, and *T* is the time window length.

**Figure 5:**
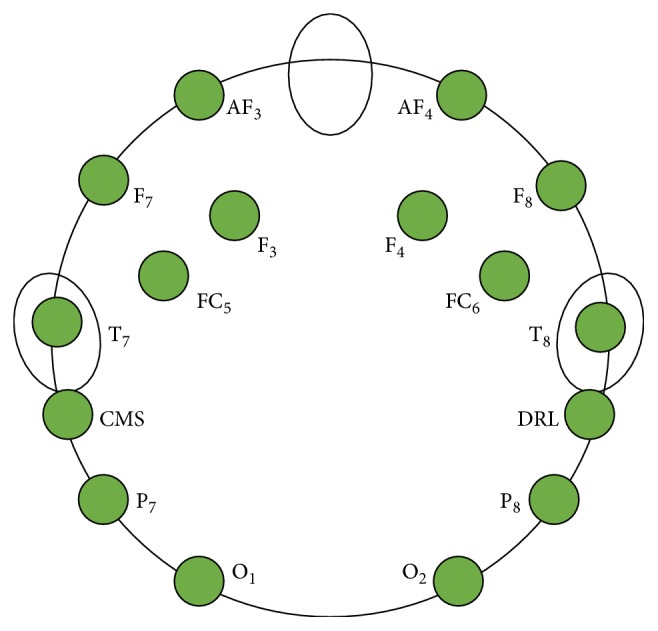
Position of EEG electrodes used in this study for recording brain and nonbrain signals.

The EEG data were sampled at a frequency of 2048 Hz and then downsampled to 128 Hz for signal processing. The electrodes were placed at 10-20 system locations, AF3, F7, F3, FC5, T7, P7, O1, O2, P8, T8, FC6, F4, F8, and AF4, as well as two reference electrodes located above the ears of the subject (i.e., either CMS and DRL or left/right mastoids). Electrodes P7, P8, O1, and O2 were selected to collect SSVEP-based EEG signal. Electrode FC5 and FC6 were used to capture EEG signals in MI. The signals during “teeth clenching” state were collected mainly by electrodes F7 and F8 (see Figure 5).

### 2.2. Processing Methods for SSVEP-Based BCI

To reduce the effect of signal-to-noise ratio (SNR), discrete wavelet transform (DWT) was employed for preprocessing of EEG signals. Assuming that *x*(*n*) is the EEG signal, the DWT of *x*(*n*) is defined as

(2) Cj,k=2−j/2∑n=−∞∞xnφ−j,k2−jn−k=xn,φj,k,j,k∈Z,

where *φ*(*n*) is the wavelet basis function, *j* is the resolution of the frequency, and *k* is the amount of time translation.

EEG signals were decomposed in different layers (5 layers) by using Daubechies wavelet (db4) function and reconstructed by removing frequency components (0–2 Hz).

The Canonical Correlation Analysis (CCA) is a multivariable statistical method, which was used to analyze the potential correlation between two sets of data [25]. CCA method has been widely used in SSVEP-based BCI system [16, 26].

Suppose two multidimensional random variables *X*, *Y*; that is, *X* ∈ *R*^*H*×*J*^, *Y* ∈ *R*^*I*×*J*^. CCA finds a pair of weight vectors *w*_*X*_ ∈ *R*^*H*×1^ and *w*_*Y*_ ∈ *R*^*I*×1^, respectively, which maximize the correlation between linear combinations *x* = *w*_*X*_^*T*^*X* and *y* = *w*_*Y*_^*T*^*Y*. It is defined as

(3) maxwX,wY⁡ρx,yExyTExxTEyyT=EwXTXYTwYEwXTXXTwXEwYTYYTwY,

where the maximum of *ρ* is the maximum canonical correlation. *x* and *y* are projected onto *w*_*X*_ and *w*_*Y*_.

The reference signals *Y*_*i*_ are set as

(4) $$\begin{array}{c} Y_{i}=\left(\begin{array}{c} \sin\left(2\pi f_{i}t\right)\\ \cos\left(2\pi f_{i}t\right)\\ \vdots \\ \sin\left(2\pi N_{h}f_{i}t\right)\\ cos\left(2\pi N_{h}f_{i}t\right) \end{array}\right),\;t=\frac{1}{S},\frac{2}{S},\ldots ,\frac{N}{S}, \end{array}$$

where *N* is the number of sampling points, *S* is the sampling frequency, and *N*_*h*_ is the number of harmonics.

The control command *K* is recognized as

(5) $$\begin{array}{c} K=\underset{i}{\max}\;\rho_{i},\;i=1,2,3,4, \end{array}$$

where *ρ*_*i*_ are the CCA coefficients obtained from the four reference signals.

Each subject has different thresholds *θ*, so threshold *θ* was defined in training phase. To calculate the threshold *θ* in CCA, 20 offline experiments were held for each subject. The results were shown in Table 2. The average correlation coefficient of idle state is 0.1542 ± 0.0397 (mean ± SD). So, the threshold *θ* was defined to be 0.22.

### 2.3. Processing Methods for MI-Based BCI and “Teeth Clenching” State Detector

The motor imagery classification based on mu frequency power has been widely used for processing event-related synchronization (ERS) and event-related desynchronization (ERD) [15, 27–29]. The second-order moment energy algorithm was employed to classify the left hand with low computational complexity and simple principle [30]. So, these algorithms could be suitable for achieving online BCI systems.

Assuming a signal of length *N*, the second-order moment is estimated by

(6) $$\begin{array}{c} E_{2}=E\left[\;x^{2}\left(\;n\;\right)\;\right]\approx \frac{1}{N}\overset{N}{\underset{n=1}{\sum }}x^{2}\left(\;n\;\right). \end{array}$$

In MI-based BCI experiment, while imagining the left hand or right hand movement, the EEG signals are collected with band-pass filtering (0–32 Hz), mu rhythm energy change of FC5 and FC6 was computed, and the energy difference between FC5 and FC6 channels is used to calculate the threshold *α* for classification. Mu rhythm energy change of FC5 and FC6 is denoted as *E*.

(7) $$\begin{array}{c} \hat{e}=\left\{\begin{array}{cc} +1 & \text{if }E>\alpha \\ 0 & \text{otherwise}\\ -1 & \text{if }E<-\alpha . \end{array}\right. \end{array}$$

When $\hat{e}=1$, which indicates the subject is imagining left hand motor imagery, the first path is closed in SPDTBS. When $\hat{e}=-1$, which indicates the subject is imagining right hand motor imagery, then the second path is closed in SPDTBS. When $\hat{e}=0$, which indicates the subject is in an idle state, no command will be given to the system, and the SPDTBS is opened.

Accuracy of detecting “teeth clenching” state is higher than other facial states in EEG-based BCI system [31]. Thus, “teeth clenching” state was detected to work as interrupt system, which can confirm motor imagery result and improve the performance of whole system.

According to the characteristics of different states (“natural” versus “teeth clenching”), the threshold *β* of standard deviation and threshold *η* of the peak distance (the absolute value of the difference between the maximum and the minimum) of the EEG signals were calculated, respectively. In online experiment, to detect the “teeth clenching” state, standard deviation *S*_*s*_ and peak distance *S*_*p*_ were computed and compared with thresholds *β* and *η*.

(8) $$\begin{array}{c} \hat{s}=\left\{\begin{array}{cc} 1 & \text{if }S_{s}>\beta ,\;S_{p}>\eta \\ 0 & \text{otherwise,} \end{array}\right. \end{array}$$

where $\hat{s}=1$ indicates that the subject is in “teeth clenching” state and, otherwise, $\hat{s}=0$ means that the subject is in “natural” state.

## 3. Results of Brain-Control Tasks

**Figure 6:**
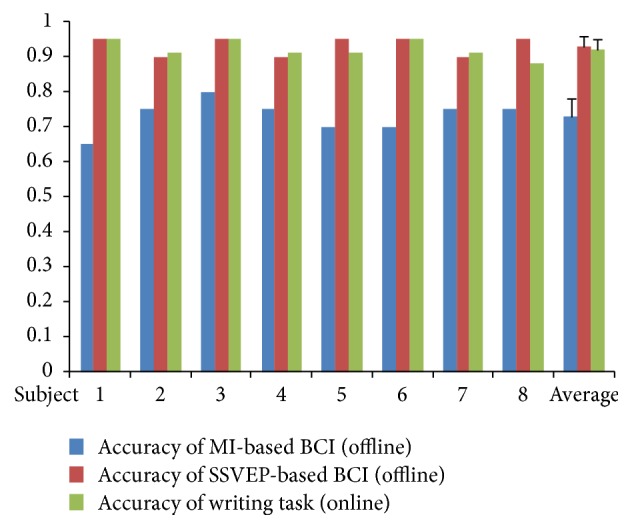
Decoding accuracy of the hybrid BCI system.

**Figure 7:**
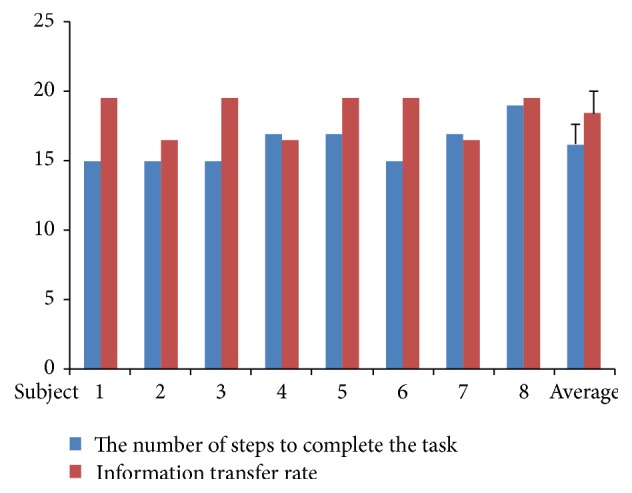
Performances of writing task.

In the first stage, an offline experiment was held for MI-based BCI and SSVEP-based BCI and “teeth clenching” state detector, respectively. As shown in Figure 6, the average accuracy of eight subjects in MI-based BCI and SSVEP-based BCI is 0.73 ± 0.05 and 0.93 ± 0.03, respectively. The ITR of SSVEP-based BCI is 18.43 ± 1.63 (Figure 7). For “teeth clenching” state detector, all subjects achieved accuracy near 1.

Eight subjects joined the writing task using a robotic arm. The results were shown in Figures 4, 6, and 7. All of subjects were successful in writing the word “HI.” Eight subjects completed the writing task in 297.37 ± 57.96 seconds on average. As shown in Figure 7, four subjects took 15 steps (optimal number of steps) to finish writing, three subjects took 17 steps, and one subject took 19 steps. Only one subject has 1 significant error. The average accuracy was obtained as 0.92 ± 0.03.

## 4. Discussion

In this paper, a novel multichannel hybrid BCI system was proposed for multidimensional control purpose, which was composed of a motor-imagery-based brain switch, “teeth clenching” state detector, and SSVEP-based BCI. For achieving a multidimensional control of robotic arm, seven commands which can be up to nine were designed for real-time BCI application. Writing task was held to evaluate the performances of the proposed hybrid system. Eight subjects completed the movement tasks of the robotic arm to write the word “HI.”

Pfurtscheller et al. [19] used MI-based brain switch to turn on/off an SSVEP BCI for reducing the errors in resting periods, while, in this present work, MI-based brain switch was used as single-pole double throw brain switch (SPDTBS) to extend the number of commands which robotic arm needs to be fully controlled and manipulated to achieve a naturalistic hand pathway of writing a simple word. In addition, a “teeth clenching” state detector was design to initialize the statement of SPDTBS. Because of the relative lower accuracy of motor imagery BCI, the “teeth clenching” state detector can confirm the result of MI-based BCI, which can improve the accuracy of hybrid BCI system significantly. Thus, the accuracy of proposed system is almost the same as previous work [19], which can lead us to conclude that the proposed system is efficient and robust for real-time multifunctional BCI systems. Compared with the current BCI systems [32–34], the proposed hybrid BCI system shows higher accuracy with high degree of freedom. Moreover, wireless manner was used to build a stable and suitable connection for online experiment, which is meaningful for portable noninvasive BCI products for real-life use.

In this study, we found that a group of healthy subjects could willingly use brain and nonbrain activity to control a robotic arm with high accuracy for performing writing tasks requiring human intention, error feedback, and multiple degrees of freedom by combination of MI, SSVEP, and EMG activity (see Supplementary video in Supplementary Material available online at https://doi.org/10.1155/2017/8316485). The robotic arm could only move and write horizontal and vertical lines using our proposed BCI paradigm. There are still two commands, “no function,” in the proposed system, which can be used for writing slanted lines. This option will be added in the near future to achieve naturalistic hand writing. Thus, the users will be able to write any intended complex word in real time, which not only can be used as communication tool for the disable people, but also can be applied for education purposes for children and students using e-learning aspect which could be an innovative way to practice using teleoperation to remotely access a robotic arm using their brain activity.

## 5. Conclusions

This paper presented a combination of synchronous and asynchronous control using a novel hybrid EEG-EMG-based BCI which consists of motor imagery, muscle artifacts, and SSVEP to provide a multidimensional control. The synchronous control is based on SSVEP paradigm which requires the user to focus on the screen and the asynchronous control is based on the motor imagery which does not need any synchronization between the user and the screen because it is based on the imagination of the unilateral movements. Users were able to write an English word using our robust real-time control of a robotic arm through the proposed hybrid BCI. This proposed BCI was designed for multiclass control in a complex environment. Results of the study indicated that successful multidimensional control is possible using suitable combination of BCI modalities to detect and classify brain activity in different situations.

In the near future, for rehabilitation, e-learning, and entertainment, we would like to design low cost, portable, noninvasive, and hybrid EEG-EMG-based robotic arm using minimum number of wearable wireless sensors.

## Supplementary Material

The video demonstrates a real-time application of our proposed hybrid BCI. This video shows a healthy subject that could willingly use his brain and nonbrain activities to control a robotic arm. The subject was able in the end to write an English work “HI” using our proposed hybrid noninvasive BCI.

## Figures and Tables

**Table 1:** The control commands of hybrid BCI.

	SPDTBS	SSVEP-based BCI frequency	Control command
Brain activity based on imagined unilateral hand movements (motor imagery) and SSVEP	Left hand imagination	6 Hz	Forward
		7.5 Hz	Backward
		8.57 Hz	Left
		10 Hz	Right
		Idle	No command
	Right hand imagination	6 Hz	No function
		7.5 Hz	No function
		8.57 Hz	Upward
		10 Hz	Downward
		Idle	No command

Muscles activity (EMG artifacts)	“Teeth clenching” state		Stop

**Table 2:** The results of canonical correlation analysis coefficients for different SSVEP states.

SSVEP state	Mean ± SD
6 Hz	0.4238 ± 0.1060
7.5 Hz	0.4621 ± 0.0857
8.57 Hz	0.4985 ± 0.1000
10 Hz	0.5105 ± 0.0381
Idle	0.1542 ± 0.0397
